# Exploring associations of anthropometric parameters and serum triglycerides with serum thyroid hormones in young women

**DOI:** 10.1038/s41598-022-22371-1

**Published:** 2022-10-17

**Authors:** Barbara Lisowska-Myjak, Hanna Zborowska, Sławomir Białek, Piotr Wroczyński, Marek Kuch, Ewa Skarżyńska

**Affiliations:** 1 Department of Biochemistry and Clinical Chemistry, Medical University of Warsaw, ul. Banacha 1, 02-097 Warsaw, Poland; 2 Department of Laboratory Diagnostics, Medical University of Warsaw, Warsaw, Poland; 3 Department of Bioanalysis and Drugs Analysis, Medical University of Warsaw, Warsaw, Poland; 4 Department of Cardiology, Hypertension and Internal Medicine, Medical Faculty, The Medical University of Warsaw, Masovian Bródnowski Hospital, Warsaw, Poland

**Keywords:** Biomarkers, Endocrinology

## Abstract

Establishing links between serum thyroid hormone panel and triglyceride (TG) concentrations with non-invasively obtained measurements of anthropometric parameters of young women may provide preliminary knowledge about the homeostasis of metabolic processes and body composition and about the strategic role of the tested parameters as early screening tests for assessing the health status of apparently healthy women in the period preceding pregnancy. The study was conducted in 381 healthy female students (aged 18–26 years, mean ± SD = 22.1 ± 1.3). Anthropometric indices (BMI, waist-to-hip ratio, FAT%) were calculated and serum concentrations of thyroid hormones (TSH, fT3, fT4) were determined using electrochemiluminescence immunoassays and serum triglycerides (TG) with a commercially available test. No association was established between serum TSH and anthropometric indices in healthy young women. Increased serum concentrations of fT4, fT3 and TG were found in overweight subjects, i.e. BMI > 24.9 kg/m^2^ (p < 0.05). A significant negative association between fT3 and TG was found in underweight subjects (r = − 0.258, p = 0.049) and a significantly positive association in normal-weight subjects (r = 0.139, p = 0.019). In healthy young women differences in BMI are not related to thyroid function. The opposite directions between the associations fT3 *vs* TG in underweight compared to normal-weight young prepregnant females may suggest dependencies of fT3 and TG in the regulation of specific BMI-dependent metabolic processes.

## Introduction

Understanding the mechanisms which underlie the associations between changes in the components of the total serum thyroid hormone concentrations and their long-term impact on body composition may offer useful screening tools to identify health problems in apparently healthy individuals^1,2^. Due to their biological properties, the effects of the thyroid hormones are both anabolic and catabolic. The thyroid hormones are involved in the regulation of thermogenesis as well as glucose and lipid metabolism and for that reason studied to explain the etiology of obesity and underweight^3^. Conversely, epidemiological and clinical studies frequently utilize anthropometric indices to differentiate and diagnose overt and subclinical thyroid function disorders. Identifying and explaining the associations between the anthropometric measurements and the thyroid hormones in apparently euthyroid young females could provide an easily available screening tool to facilitate early detection of a variety of metabolic disorders as well as identify the risks for development of cardiovascular disorders, chronic disease, depression and reproductive hormone disorders^4–12^.

Young women in the prepregnancy period are at especially high risk for thyroid disorders which may lead to further aggravation of pre-existing chronic thyroid disease, and complications during pregnancy in the future^13^.

Commonly used anthropometric measurements such as weight, height, waist and hip circumferences and two indicators of body fatness, body mass index (BMI), a measure of weight adjusted for height and waist-to-hip ratio (WHR) are easy to obtain and give a picture of an individual’s health, but when combined with thyroid function parameters may signal the risk of long-term impact of thyroid dysfunction leading to complex health problems^14–18^.

Dyslipidemia significantly and independently associated with thyroid dysfunction is also a common feature in euthyroid populations. Hypertriglyceridemia is next to BMI a common feature in obesity indicating the accumulation of triglycerides in different tissues and associated with ectopic fat obesity. Serum levels of triglycerides can be increased in hypothyroidism and the reverse is observed in hyperthyroidism indicating the potential effect of triglyceride regulation by impaired thyroid function^12,19–22^.

The aim of the study was to explore the possible associations between serum concentrations of the thyroid hormones, i.e. thyroid stimulating hormone (TSH), free thyroxine (fT4), free triiodothyronine (fT3), and fT3/fT4 ratio, selected anthropometric indices (BMI, WHR, FAT%) and serum triglycerides (TG) in young apparently healthy prepregnant women.

## Material and methods

### Subjects

The subjects were 381 healthy unrelated female students aged 18 to 26 years (mean age ± SD = 22.1 ± 1.3), participating in the “Cholesterol Alert” program at the Medical University of Warsaw from March 2013 to July 2014.

Medical history, including a medication history, was obtained by the examining physicians to exclude cardiovascular disorders, inflammation, diabetes mellitus, malignancy, autoimmune disease, thyroid disease, thyroid supplementation or antithyroid treatment, hypolipidemic treatment (e.g. statins), and pregnancy. All participants underwent routine medical examination, had anthropometric measurements and provided overnight fasting blood samples.

### Anthropometric measurements

*Body weight* was measured in subjects wearing light clothing and without shoes and recorded to the nearest 0.1 kg.

*Height* was measured using a stadiometer in subjects without shoes and recorded to the nearest 1.0 cm.

*Body Mass Index (BMI)* [kg/m^2^] was calculated by dividing weight (kg) by the square of height (m^2^). Subjects were classified by the BMI in accordance with the WHO guidelines^23^ as follows: Underweight—BMI less than 18.5 (n = 59/381; 15.5%), Normal weight—BMI 18.5 to 24.9 (285/381, 74.8%), Overweight—BMI 25 to 29.9 (28/381, 7.3%), Obesity class I—BMI 30.0 to 34.9 (n = 8), Obesity class II—BMI 35.0 to 9.9 (n = 1).

*Waist circumference* was measured in the horizontal plane at the midpoint between the lowest ribs and the iliac crest, to the nearest 1 cm.

*Hip circumference* was measured around the widest portion of the buttocks, to the nearest 1 cm.

*Waist-to-hip ratio (WHR)* is the ratio of the circumference of the waist [cm] to that of the hips [cm].

*Percentage of body fat (FAT %)* was measured with the Tanita BC 420MA analyzer (Tanita Corporation, Tokyo, Japan).

### Laboratory methods

Serum TSH, fT3 and fT4 were measured using electrochemiluminescence immunoassays (Roche Diagnostics, Mannheim, Germany) The normal reference ranges: TSH 0.4–4.0 mU/L; fT4 9.0–25.0 pmol/L; fT3 3.5–7.8 pmol/L.

Serum triglycerides (TG) were measured using a commercially available test on the ADVIA Centaur XP System (Siemens Healthcare Diagnostics).

### Material

Blood samples were collected after overnight fast of at least eight hours. In total, 381 blood samples were drawn by venipuncture into test tubes which did not contain an anticoagulant and allowed to clot at room temperature. After centrifugation at 3000*g* for 10 min at 4 °C the serum was extracted immediately and stored at – 80 °C until assayed. On the day of the measurements serum samples were thawed at room temperature using gentle vortexing.

### Statistical analysis

In order to examine the relationship between thyroid parameters, anthropometric indices and serum TG concentrations, statistical analysis softwares such as JAMOVI and IBM SPSS Statistics 25 were used.

Descriptive statistics in two groups were isolated in regard to the level of TSH, whereas the value of 2.5 mU/L was considered as threshold between high (> 2.5 mU/L) and low (< 2.5 mU/L) concentration of TSH. The normality of dependent variables in subgroups was estimated with the use of skewness, kurtosis and Kolmogorow–Smirnow normality test.

Subsequently, to analyze the differences in dependent variables between two TSH concentration groups and taking into consideration the values of BMI, parametric MANCOVA (*Multivariate Analysis of Variance*) was used. Before the proper analysis, the Mahalanobis distance was calculated, in order to detect multivariate outliers. As cutoff point, the value of 2 standard deviations from the mean was used.

As the group sizes (*n*_*1*_ = 232, *n*_*2*_ = 143 for low and high concentration of TSH respectively) were big enough, according to the central limit theorem, the assumption of normality of means’ sampling distribution in MANCOVA was fully fullfilled. The homogeneity of variances was checked multivariably with Box’s test of equality of covariances matrices and univariably with Levene’s test of equality of error variances. The result of Box’s test was statistically significant (*F* = 1.84; *df*_*1*_ = 36; *df*_*2*_ = 309,321.12; *p* = 0.002), however only one of Levene’s tests appeared to be statistcally significant (variable TG: *F* = 5.86; *df*_*1*_ = 1; *df*_*2*_ = 373; *p* = 0.016). Taking into account the high power of Box’s test, it was considered, that the assumption was not considerably violated. However, to counteract the potentially adverse effects of light violation, the Pillai’s trace robust test statistics was used in multivariate MANCOVA. As the effect size coefficient, partial *η*^2^ was used. After omnibus MANCOVA, simple univariate between-subject effects tests were conducted. The global significance level α was set to 0.05.

To examine the dependencies of BMI on thyroid parameters, anthropometric indices and serum TG concentrations, the subjects were classified by BMI as underweight, normal weight and overweight. Serum thyroid hormones (TSH, fT3, fT4, fT3/fT4), anthropometric parameters (BMI, waist circumference, hip circumference, WHR, FAT%) and TG serum concentrations were adjusted for BMI and TSH and compared using ANOVA. Data are presented within each group as mean ± standard deviation (SD), median, range, coefficient of variation using the STATISTICA version 13.3 software http://www.statsoft.com, with the assumed statistical significance threshold of p ≤ 0.05. For the study of the relationship between BMI and selected parameters, one-way ANOVA was used with post-hoc Games-Howell and Tukey tests.

### Ethical approval

The study protocol was reviewed and approved by The Bioethics Committee of the Medical University of Warsaw, Number KB/258/2012, December 11th 2012. All methods were performed in accordance with the relevant guidelines and regulations.

### Consent to participate

All participants signed an informed consent form after receiving an explanation of study’s objectives and methodology.

## Results

Table 1 assesses the differences in dependent variables between the two groups of TSH concentrations, taking into account BMI values, in relation to high TSH levels (> 2.5 mU/L) and low TSH levels (< 2.5 mU/L). The analysis was carried out with the MANCOVA Omnibus parametric test for each effect.

**Table 1:** The results of Pillai’s trace omnibus MANCOVA tests for two main effects: one factor (TSH) and one covariate (BMI).

Effect	*V*	*F*	*df*_*1*_	*df*_*2*_	*p*	Partial *η*^2^
BMI	0.75	139.55	8	365	< 0.001	0.75
TSH	0.12	6.01	8	365	< 0.001	0.12

*V* Pillai's trace test statistics.

Both effects were statistically significant and the effect size coefficient was big for BMI and moderate for TSH groups.

Table 2 shows univariate comparisons between the two groups of TSH (TSH < 2.5 mU/L compared to > 2.5 mU/L).

**Table 2:** The results of single between-subject effects tests for TSH factor.

Dependent Variable	TSH ≤ 2.5	TSH > 2.5	*MS*	*F*	*p*	Partial *η*^2^
fT4 [pmol/l]	16.57 ± 2.32	16.82 ± 2.64	5.29	0.88	0.348	0.00
fT3 [pmol/l]	4.80 ± 0.69	5.15 ± 0.65	11.27	25.03	< 0.001	0.06
fT3/fT4	0.29 ± 0.04	0.31 ± 0.4	0.03	15.65	< 0.001	0.04
Waist circumference [cm]	72.55 ± 7.33	72.02 ± 6.23	8.03	0.43	0.515	0.00
Hip circumference [cm]	95.62 ± 6.68	96.12 ± 6.68	50.64	2.65	0.105	0.01
WHR	0.76 ± 0.05	0.75 ± 0.05	0.01	2.86	0.091	0.01
FAT [%]	22.46 ± 6.15	22.59 ± 6.58	10.92	0.86	0.355	0.00
TG [mmol/l]	0.78 ± 0.35	0.89 ± 0.414	104.46	8.97	0.003	0.02

W*HR* waist hips ratio, data in TSH columns were presented as mean ± standard deviation.

A significant increase in fT3, fT3/fT4 and TG in the TSH range > 2.5 mU/L was demonstrated. There were no significant differences (p > 0.05) in waist circumference, hip circumference, WHR and FAT [%] between the two TSH groups. The effect size coefficients indicated moderate and two small effects.

For BMI six out of eight univariate tests appeared to be statistically significant (Table 3).

**Table 3:** The results of single between-subject effects tests for BMI covariate.

Dependent variable	*B*	Pearson's *r*	*MS*	*F*	*p*	Partial *η*^2^
fT4 [pmol/L]	− 0.05	− 0.04	6.43	1.07	0.301	0.00
fT3 [pmol/L]	0.02	0.13*	1.64	3.64	0.057	0.01
fT3/fT4	0.00	0.16**	0.02	8.51	0.004	0.02
Waist circumference [cm]	1.93	0.78***	10,905.80	577.67	< 0.001	0.61
Hip circumference [cm]	1.80	0.74***	9526.60	497.97	< 0.001	0.57
WHR	0.01	0.29***	0.09	40.86	< 0.001	0.10
FAT [%]	1.86	0.82***	10,146.63	795.67	< 0.001	0.68
TG [mmol/dL]	2.34	0.20***	180.62	15.51	< 0.001	0.04

*WHR* waist hips ratio, *p < 0.05, **p < 0.01, ***p < 0.001.

The relationship between BMI and waist circumference, hip circumference and percentage of fat respectively, could be described as strongly positive. For remaining three parameters (fT3/fT4, WHR and TG) it is weak and also positive. It means, that higher values of BMI are connected with higher values of these parameters.

The serum concentrations of the thyroid hormones (TSH, fT4, fT3, fT3/fT4), anthropometric parameters (waist circumference, hip circumference, WHR, FAT[%]) and serum TG concentrations, were stratified by BMI [kg/m^2^] range into underweight, normal weight and overweight groups in the study population of 381 young healthy women (Table 4). Subsequently, in each BMI-related group associations were explored between the thyroid hormones, anthropometric measurements, and serum TG concentrations.

**Table 4:** Characteristics of the study population.

Parametrs Mean ± SD; median (range), *CV*	BMI ranges [kg/m^2^]			ANOVA *p*-value
	Underweight BMI < 18.5	Normal weight BMI = 18.5–24.9	Overweight^#^ BMI > 25	
n	**59**	**285**	**37**	
TSH [mU/L]	2.25^a^ ± 0.97; **2.15** (0.44–4.63), *43.21*	2.59^a^ ± 2.81; **2.17** (0.29–40.40), *108.15*	2.58^a^ ± 1.14; **2.15** (0.76–5.58), *44.16*	0.614
FT4 [pmol/L]	16.89^a^ ± 2.52; **17.00** (10.50–22.50), *14.94*	16.72^a^ ± 2.62; **16.60** (8.78–31.80), *15.68*	16.11^a^ ± 2.09; **16.10** (10.60–20.70), *12.98*	0.324
FT3 [pmol/L]	4.87^a^ ± 0.70; **4.80** (2.82–6.54), *14.34*	4.91^a^ ± 0.68; **4.94** (2.88–7.88), *13.84*	5.23 ± 0.76; **5.11** (3.52–7.07), *14.61*	0.025
fT3/fT4	0.29 ± 0.04; **0.29** (0.20–0.38), *15.39*	0.29 ± 0.04; **0.29** (0.16–0.48), *14.59*	0.33 ± 0.04; **0.32** (0.25–0.42), *13.63*	< 0.001
Waist cicumference [cm]	65.9 ± 4.0; **66.0** (57.0–77.0), *6.1*	72.2 ± 5.2; **72.0** (61.0–88.0), *7.2*	85.4 ± 10.6; **84.0** (71.0–128.0), *12.4*	< 0.001
Hip circumference [cm]	89.3 ± 4.2; **89.0** (75.0–99.5), *4.8*	95.7 ± 5.7; **96.0** (62.0–109.0), *6.0*	107.3 ± 8.6; **106.0** (95.0–145.0), *8.0*	< 0.001
WHR	0.74^a^ ± 0.05; **0.74** (0.65–0.83), *6.13*	0.76^a^ ± 0.06; **0.75** (0.65–1.27), *7.44*	0.79 ± 0.05; **0.78** (0.71–0.89), *6.04*	< 0.001
FAT%	14.6 ± 3.7; **14.5** (5.8–22.1), *25.3*	22.9 ± 4.5; **22.8** (9.1–34.1), *19.7*	33.1 ± 6.0; **32.6** (19.3–50.5), *18.0*	< 0.001
TG [mmol/L]	0.76^a^ ± 0.25; **0.75** (0.38–1.60), *32.8*	0.81^a^ ± 0.36; **0.71** (0.27–2.42), *44.1*	1.05 ± 0.55; **0.93** (0.35–3.04), *52.1*	0.015

Group means with the same letter indices do not differ significantly from each other, p < 0.050, Tukey HSD post-hoc for equal variances and Games-Howell test for unequal variances.

Median values are in bold, CV in italic style.

^**#**^Overweight refers to both and overweight subjects + obesity class I + obesity class II (n = 28 + n = 8 + n = 1) *CV* = coefficient of variation.

In overweight subjects (BMI > 25) serum fT3, fT3/fT4 ratio and TG levels were higher than in underweight and normal-weight subjects (p < 0.05). There were no significant differences in these parameters between underweight and normal-weight subjects (p > 0.05). TSH concentrations did not significantly differ between the three groups. The anthropometric measurements (waist and hip circumferences, WHR, FAT%) consistently increased with each BMI range. For consecutive BMI ranges differences (p < 0.05) were found in the associations between the serum TG concentrations and the thyroid hormones and anthropometric measurements. In underweight subjects a negative correlation was established between TG *vs* fT4 and TG *vs* fT3 (r = − 0.306, − 0.258, respectively), in normal-weight subjects a positive correlation was established between TG *vs* TSH, TG *vs* fT3, TG *vs* fT3/fT4, TG *vs* FAT%, (r = 0.181, 0.139, 0.138, 0.117, respectively), while in the overweight range there was no association between these parameters.

**Figure 1:**
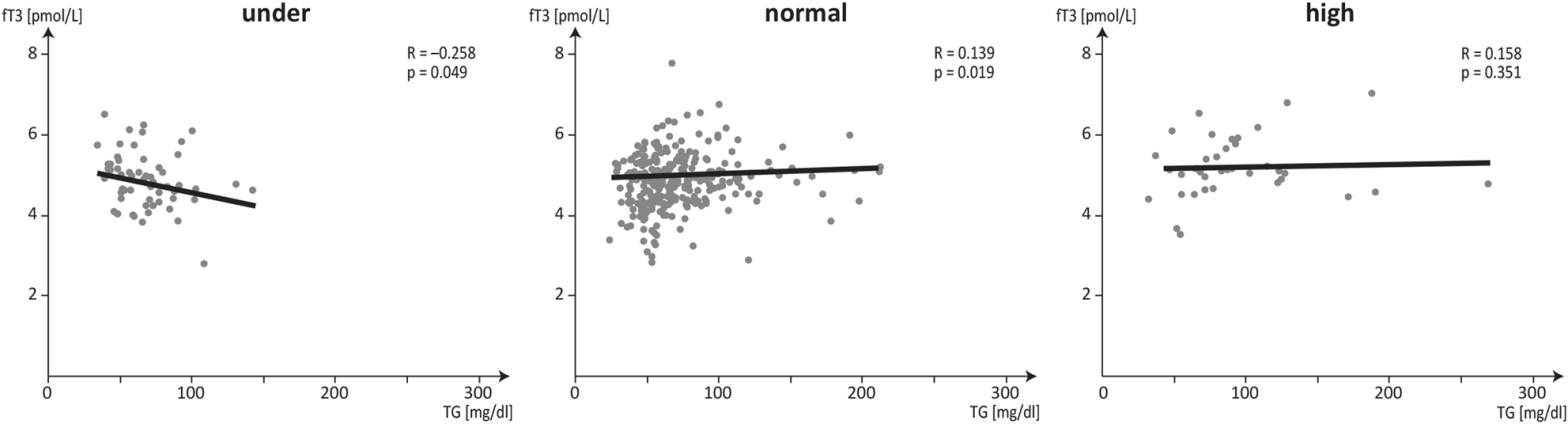
Changing correlations between fT3 and TG serum concentrations in healthy young underweight, normal-weight and overweight women.

The differences between the correlations of fT3 and TG concentrations within the BMI ranges (underweight, normal and overweight) are presented on the graph in Fig. 1.

**Figure 2:**
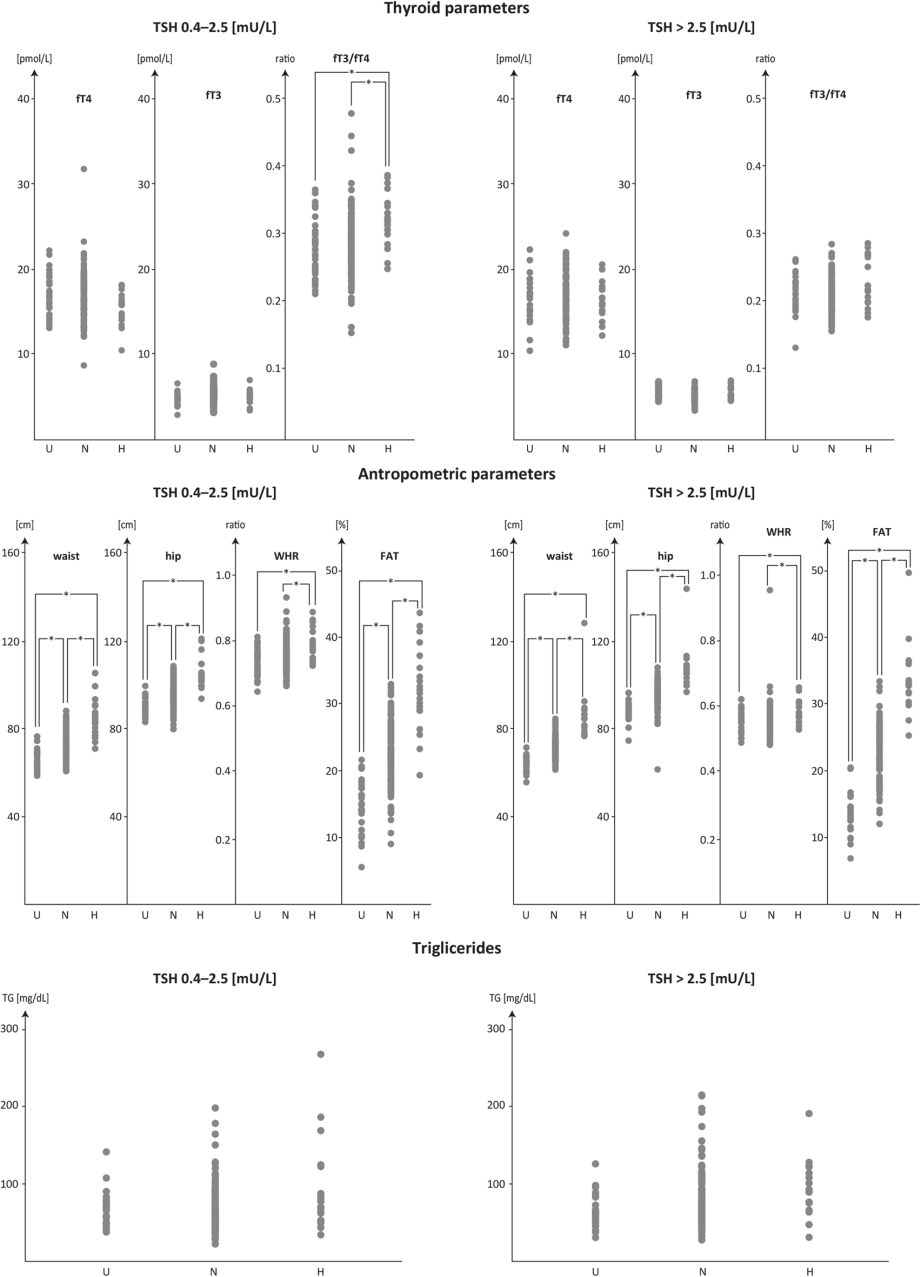
Effects of TSH > 2.5 mU/L on serum concentrations of thyroid hormones (fT4, fT3, fT3/fT4), anthropometric parameters (waist circumference, hip circumference, WHR, FAT%) and serum triglyceride concentrations.

The differences in the thyroid and anthropometric parameters and serum TG measured in underweight, normal-weight and overweight subjects within the Normal vs High TSH groups (TSH ranges 0.4 to 2.5 mU/L vs > 2.5 mU/L) are presented on the graph in Fig. 2.

An increase in the fT3/fT4 ratios (p < 0.05) found in the underweight *vs* overweight subjects and the normal-weight *vs* overweight subjects occurred within the normal TSH range (0.4 to 2.5 mU/L). An increase in anthropometric parameters found in the underweight *vs* normal weight *vs* obese subjects was observed within both normal and high TSH ranges. No association was established between the TSH range and serum TG concentrations in underweight, normal-weight and overweight subjects.

## Discussion

The above results showed characteristic differences in the interrelations of serum fT3 vs TG between young healthy women who were underweight, normal body weight, and overweight. The search for such links is a consistent response to the demand of clinicians who see the diagnostic potential to explain the relationship between somatic parameters and health status as an important public health problem^1,4,8,9,24^.

Young apparently healthy women in the prepregnancy period of their lifecycle are a population at risk of early thyroid dysfunction and abnormal anthropometric measurements with long-term adverse health effects such as an increased incidence of metabolic disorders, cardiovascular disorders, chronic diseases, depression, and age-specific reproductive hormone disorders with a range of pre-pregnancy complications^11,13,16,24–27^. The symptoms of altered thyroid function are often non-specific, may differ widely and be difficult to identify. While thyroid dysfunction may occur in asymptomatic patients, the altered concentrations of the thyroid hormones can adversely affect virtually every organ system of the body^28^. Both underweight and overweight can have adverse effects on female reproductive health, including an increased risk for menstrual dysfunction, anovulatory infertility and other reproductive disorders^25,29^. Maternal underweight in early pregnancy may lead to suppressed fetal development, resulting in decreased birthweight and according to the “fetal origins of adult diseases” hypothesis is associated with a greater risk for the later development of ischemic heart disease and chronic diseases in the offspring^27,30,31^. Maternal overweight and obesity are risk factors for gestational diabetes and hypertensive disorders of pregnancy and are linked to an increased risk of macrosomia^27^.

The thyroid hormone concentrations, anthropometric indices and serum TG were compared between underweight, normal-weight and overweight subjects to explore interaction involved in the regulation of the thyroid hormones and somatic parameters in a selected population of young apparently healthy women. As reported in the literature^11^, the values of serum fT3, fT3/fT4 and TG were higher in overweight subjects compared to normal-weight women, but unlike other authors we did not observe changes in the serum concentrations of TSH and fT4 with increasing BMI. That could result from a small number of obese subjects (BMI > 29.9) included in the study. Despite significant differences in the anthropometric indices, there were no differences in the serum concentration of the thyroid hormones and TG between underweight and normal weight subjects.

The presented results join the important discussion for clinical decisions whether altered serum concentrations of thyroid hormones are a direct result of thyroid disease or whether they can be secondary to altered body weight^4,8,9,11,29^. The presented results do not show significant differences between TSH and fT4 concentrations in subsequent BMI ranges.

In the study, serum TSH measurements were used as the most common, specific and sensitive laboratory marker of primary thyroid disease^32^. As seen in the literature, reference TSH ranges remain controversial and there is no undisputed universal reference range for women with normal thyroid function, taking into consideration that the ‘normal’ TSH level may depend on a person’s age, ethnicity, iodine intake, time of day when a blood sample is collected or the type of TSH assay. The high susceptibility of the body's biological functions to the influence of thyroid hormones may suggest that even small fluctuations in the concentrations of these hormones can cause clinical symptoms that are difficult to interpret.

Clinicians emphasize the need to determine the ‘true normal’ TSH range in healthy young women to aid in the early detection of minimally altered thyroid function outside normal ranges, which is considered a risk factor contributing to the worsening of metabolic complications and development of disease in the thyroid^3,4,6,10,11,31,33^. The commonly accepted typical reference range for TSH concentrations is between 0.2–0.4 and 4.0–4.5 mU/L, but increasingly more authors are in support of the lower cut off value of 2.5 mU/L of upper normal limit of TSH to identify thyroid dysfunction^11,32^. Given that the initial criteria qualified the young women to the healthy group, the question arises which of the presented cut-off values may be more effective for diagnosing latent thyroid diseases and their biological effects in this age group. According to the opinion of some authors, the TSH cut-off value of > 4.0 mU/L is sufficient for the selection of impaired thyroid function and is commonly used in clinical routine^34^. On the other hand, an increase in TSH > 2.5 mU/L allows for a more sensitive distinction of young women during the pregnancy planning period, with an indication of an increase in the risk of health disorders in later life and during pregnancy^5,8,9^. Normal TSH (< 2.5 mU/l) was in 62% of subjects and high TSH (> 2.5 mU/L) in 38%. The pathomechanism of selecting such a large number of young women based on TSH > 2.5 mU/L values is unclear. It seems that a further diagnostic strategy of these women requires the choice of additional laboratory and clinical parameters.

Despite numerous experimental studies and clinical observations, the mechanism of long-term impact of BMI changes on thyroid dysfunction remains unclear^6–9,11,25^. The absence of significant differences in TSH concentrations in subsequent BMI ranges in healthy young women does not support the common opinion of a direct association between hypothyroidism and obesity or between hyperthyroidism and weight loss^3^. However, the results can demonstrate local involvement of fT3 and fT3/fT4 ratio in the regulation of body weight and confirm another opinion that changes in the serum concentrations of these hormones may be a consequence rather than the cause of changes in the body weight^10,35^. Free triodothyronine (fT3) is the active form, converted from free thyroxine (fT4) in peripheral tissues, and the degree of conversion is reflected in the fT3/fT4 ratio. Local control of thyroid hormones by different deiodinases in the context of the tissue-specific impact on energy metabolism and the metabolic functions remains to be further elucidated^13,17^.

Considering that thyroid hormones are involved in multiple physiological processes and regulate basal metabolic rate, generate heat, stimulate gluconeogenesis and both lipolysis and lipogenesis, their activities may be reflected not just in their blood levels, but also in biological effects. Understanding of the causal relationship between thyroid hormone concentrations and various metabolic indices can be of crucial importance for a range of diagnostic and therapeutic decisions. As reported in the literature, potential mechanisms underlying the effect of changes in thyroid hormones on lipid metabolism, including TG as the most common type of fat in the body have been demonstrated in experimental animal studies, advances in molecular biology and clinical studies^8,9,11,21^. The associations between serum concentrations of fT3 and TG demonstrate the differences between, underweight, normal-weight and overweight young females. Lack of an evident association between the serum concentrations of fT3 and TG in overweight subjects remains unexplained although several mechanisms have been proposed to account for changes in thyroid hormone concentrations in obesity, including adaptive thermogenesis to increase energy expenditure, hyperleptinemia, altered peripheral deiodinase activity, thyroid hormone resistance, chronic low-grade inflammation and insulin resistance^7,11,17^. The characteristic associations between TG and fT3, negative in underweight young women and positive in normal weight women, may suggest different active involvement of fT3 and TG in the regulation of specific BMI-dependent metabolic processes. Further studies are needed to explore the causal relationship between fT3 and TG in young underweight women and its biological effects on health, both short- and long-term, the latter to help the body prepare itself for pregnancy and avoid adverse outcomes in the prepregnancy period.

To conclude, in healthy young women BMI does not depend on altered thyroid function. BMI changes can be induced by the local fT3 activities and the fT3/fT4 ratio which are involved in the regulation of specific metabolic processes. The observation that the associations between fT3 and TG in underweight *vs* normal-weight young prepregnant females move in the opposite directions may suggest active involvement of fT3 and TG in the regulation of specific BMI-dependent metabolic processes.

## Data Availability

The datasets used and/or analysed during the current study available from the corresponding author on reasonable request.

## References

[1] Do JG, Park CH, Lee YT, Yoon KJ (2019). Association between underweight and pulmonary function in 282,135 healthy adults: A cross-sectional study in Korean population. Sci. Rep..

[2] Sakamoto A, Yanagimoto S, Tanaka K, Komuro I, Koike K (2019). Abstract 10373: Low body mass index independently predicts future risk of elevated low-density lipoprotein cholesterol levels in apparently healthy women. Circulation.

[3] Fontenelle LC, Feitosa MM, Severo JS, Freitas TEC, Morais JBS, Torres-Leal FL, Henriques GS, do Nascimento Marreiro D (2016). Thyroid function in human obesity: Undrerlying mechanisms. Horm. Metab. Res..

[4] Chin KY, Ima-Nirwana S, Mohamed IN, Aminuddin A, Johari MH, Ngah WZW (2014). The relationships between thyroid hormones and thyroid-stimulating hormone with lipid profile in euthyroid men. Int. J. Med. Sci..

[5] Nie X, Xu Y, Ma X, Xiao Y, Wang Y, Bao Y (2020). Association between abdominal fat distribution and free triiodothyronine in a euthyroid population. Obes. Facts.

[6] Nowak M, Kalwa M, Oleksy P, Marszałek K, Radon-Pokracka M, Huras H (2019). The relationship between pre-pregnancy BMI, gestational weight gain and neonatal birth weight: A retrospective cohort study. Gin. Pol..

[7] Őzer S, Bűtűn I, Sőnmezgőz E, Yilmaz R, Demir O (2015). Relationships among thyroid hormones and obesity severity, metabolic syndrome and its components in Turkish children with obesity. Nutr. Hosp..

[8] Park D, Lee JH, Han S (2017). Underweight: Another risk factor for cardiovascular disease?. Medicine.

[9] Park SY, Park SE, Jung SW, Jin HS, Park IB, Ahn SV, Lee S (2017). Free triiodothyronine/free thyroxine ratio rather than thyrotropin is more associated with metabolic parametres in healthy euthyroid adult subjects. Clin. Endocrinol..

[10] Soriguer F, Valdes S, Morcillo S, Esteva I, Almaraz C, de Adana MSR, Tapia MJ, Dominguez M, Gutierrez-Repiso C (2011). Thyroid hormone levels predict the change in body weight: A prospective study. Eur. J. Clin. Investig..

[11] Xu R, Huang F, Zhang S, Lv Y, Liu Q (2019). Thyroid function, body mass index, and metabolic risk markers in euthyroid adults: A cohort study. BMC Endocr. Disord..

[12] Zhang X, Chen W, Shao S, Xu G, Song Y, Xu C, Gao L, Hu C, Zhao J (2018). A high-fat diet rich in saturated and mono-unsaturated fatty acids induces disturbance of thyroid lipid profile and hypothyroxinemia in male rats. Mol. Nutr. Food Res..

[13] Lisowska-Myjak B, Puchalska A, Hałasa N, Płazińska M, Strawa A (2019). The association between clinical and laboratory paramete rs in thyroid disease and nonthyroidal illness in young women. Eur. Rev. Med. Pharmacol. Sci..

[14] Cicatiello AG, Di Girolamo D, Dentice M (2018). Metabolic effects of the intracellular regulation of thyroid hormone: Old players, new concepts. Front. Endocrinol..

[15] de Morais NAO, de Assis ASA, Corcino CM, Saraiva DA, Berbara TMBL, Ventura CDD, Vaisman M, Teixeira PFS (2018). Recrent recommendations from ATA guidelines to define the upper reference range for serum TSH in the first trimester match reference ranges for pregnant women in Rio de Janeiro. Arch. Endocrinpol. Metab..

[16] Fatourechi V (2009). Subclinical hypothyroidism: An update for primary care physicians. Mayo Clin. Proc..

[17] Nam JS, Cho M, Park JS, Ahn CW, Cha BS, Lee EJ, Lim SK, Kim KR, Lee HC (2010). Triiodothyronine level predicts visceral obesity and atherosclertosis in euthyroid, overweight and obese subjects: T3 and visceral obesity. Obesity Res. Clin. Pract..

[18] Shibata Y, Ojima T, Nakamura M, Kuwabara K, Miyagawa N, Saito Y, Nakamura Y, Kiyohara Y, Nakagawa H, Fujiyoshi A, Kadota A, Ohkubo T, Okamura T, Ueshima H, Okayama A, Miura K (2019). Associations of overweight, obesity, and underweight with high serum total cholesterol level over 30 years among the Japanese eldery: NIPPON DATA 80, 90, and 2010. J. Epidemiol..

[19] Sinha RA, Singh BK, Yen PM (2018). Direct effects of thyroid hormones on hepatic lipid metabolism. Nat. Rev. Endocrinol..

[20] Yang J, Zhou X, Zhang X, Hu J, Gao L, Song Y, Yu C, Shao S, Yuan Z, Sun Y, Yan H, Li G, Zhao J (2016). Analysis of the correlation between lipotoxicity and pituitary-thyroid axis hormone levels in men and male rats. Oncotarget.

[21] Zhao M, Tang X, Yang T, Zhang B, Guan Q, Shao S, Xue F, Zhang X, Liu Z, Yuan Z, Song Y, Zhang H, Fang L, Yu C, Li Q, Zhang X, Gao L, Xu C, Zhao J (2015). Lipotoxicity, a potential risk factor for the increasing prevalence of subclinical hypothyroidism?. J. Clin. Endocrinol. Metab..

[22] Zou Y, Sheng G, Yu M, Xie G (2020). The association between triglycerides and ectopic fat obesity: An inverted U-shaped curve. PLoS ONE.

[23] Weir, C. B. & Jan, A. BMI classification percentile and cut off points [Updated 2020 Jul 10]. In *StatPearls* (StatPearls Publishing, 2021). 31082114

[24] Nilsson G, Hedberg P, Leppert J, Ohrvik J (2018). Basic anthropometric measures in acute myocardial infarction patients and individually sex- and age-matched controls from the general population. J. Obes..

[25] Mahutte N, Kamga-Ngande C, Sharma A, Sylvestre C (2018). Obesity and reproduction. J. Obstet. Gynaecol. Can..

[26] Ringbäck Weitoft G, Eliasson M, Rosén M (2008). Underweight, overweight and obesity as risk factors for mortality and hospitalization. Scand J. Public Health..

[27] Suastika K, Dwipayana P, Saraswati MR, Gotera W, Budhiarta AAG, Sutanegara ND, Gunadi GNP, Nadha KB, Wita W, Rina K, Santoso A, Soegondo S, Kajiwara N, Taniguchi (2011). Underweight is an important risk factor for coronary heart disease in the population of Ceningan Island, Bali. Diabetes Vasc. Dis. Res..

[28] Lahoti A, Frank GR (2013). Laboratory thyroid function testing: Do abnormalities always mean pathology?. Clin. Ped..

[29] Boutari C, Pappas PD, Mintziori G, Nigdelis MP, Athanasiadis L, Goulis DG, Mantzoros CS (2020). The effect of underweight on female and male reproduction. Metab. Clin. Exp..

[30] Huxley R, Owen CG, Whincup PH, Cook DG, Rich-Edwards J, Smith GD, Collins R (2007). Is birth weight a risk factor for ischemic heart disease in later life?. Am. J. Clin. Nutr..

[31] Szostak-Węgierek D, Waśkiewicz A, Piotrowski W, Stepankiak U, Pająk A, Kwaśniewska M, Nadrowski P, Niklas A, Puch-Walczak A, Dryglas W (2018). Metabolic syndrome and its components in Polish women of childbearing age: A nationwide study. BMS Public Health.

[32] Lewandowski K (2021). Reference ranges for TSH and thyroid hormones. Thyroid Res..

[33] Rios-Prego M, Anibarro L, Sánchez-Sobrino P (2019). Relationship between thyroid dysfunction and body weight: A not so evident paradigm. Int. J. Gen. Med..

[34] Arce-Sánchez L, Vitale SG, Flores-Robles CM, Godines-Enriquez MS, Noventa M, Urquia-Figueroa CM, Martínez Cruz N, Estrada-Gutierrez G, Espino y Sosa S, Romo-Yañez J, Montoya-Estrada A, Reyes-Muñoz E (2021). Effect of the cut-off level for thyroid-stimulating hormone on the prevalence of subclinical hypothyroidism among infertile Mexican women. Diagnostics.

[35] Punda A, Škrabić V, Torlak V, Gunjača I, Perica IK, Polašek O, Hayward C, Zemunik T, Matana A (2020). Thyroid hormone levels are associated with metabolic components: A cross-sectional study. Croat. Med. J..

